# Do not Lose Your Students in Large Lectures: A Five-Step Paper-Based Model to Foster Students’ Participation

**DOI:** 10.3390/pharmacy3030089

**Published:** 2015-07-27

**Authors:** Mona Hassan Aburahma

**Affiliations:** Department of Pharmaceutics and Industrial Pharmacy, Faculty of Pharmacy, Cairo University, Kasr El-Aini Street, Cairo 11562, Egypt; E-Mail: mona_aburahma@hotmail.com

**Keywords:** large lectures, paper-based approach: economic constrains, active learning, feedback, writing

## Abstract

Like most of the pharmacy colleges in developing countries with high population growth, public pharmacy colleges in Egypt are experiencing a significant increase in students’ enrollment annually due to the large youth population, accompanied with the keenness of students to join pharmacy colleges as a step to a better future career. In this context, large lectures represent a popular approach for teaching the students as economic and logistic constraints prevent splitting them into smaller groups. Nevertheless, the impact of large lectures in relation to student learning has been widely questioned due to their educational limitations, which are related to the passive role the students maintain in lectures. Despite the reported feebleness underlying large lectures and lecturing in general, large lectures will likely continue to be taught in the same format in these countries. Accordingly, to soften the negative impacts of large lectures, this article describes a simple and feasible 5-step paper-based model to transform lectures from a passive information delivery space into an active learning environment. This model mainly suits educational establishments with financial constraints, nevertheless, it can be applied in lectures presented in any educational environment to improve active participation of students. The components and the expected advantages of employing the 5-step paper-based model in large lectures as well as its limitations and ways to overcome them are presented briefly. The impact of applying this model on students’ engagement and learning is currently being investigated.

## 1. Introduction

In the Middle East, Egypt pioneered in introducing pharmacy education, which started at Cairo University in 1824. Although Egypt has the largest number of pharmacy colleges in the Middle East, either public or private, that results in more than 13,000 pharmacist graduates annually [1], pharmacy colleges are still experiencing significant growth in students’ enrollments annually. Accordingly, lecturers in the pharmacy colleges are forced to rely on traditional lectures that span typically for 1 h for one-way transmission of factual information to passive students. The wide spread of large lectures in the Egyptian public universities represents an economic necessity born due to resource restrictions associated with the large number of students.

In educational literature, there is great discrepancy in defining the term “large lectures” [2], nevertheless, in this article, the large lecture is a representation of any lecture that contains more than 100 students.

Generally speaking, lectures are ideal for presenting information or delivering educational content to a large number of students in a cost-effective way [3]. In addition, lectures permit maximum teacher control regarding the pacing and range of material presented. In relation to students, lectures present the least threat to students, since they are not obliged to participate in comparison to workshops or tutorials [3]. In addition, lectures represent a consistent learning experience for all students [4].

On the other hand, large lectures suffer from significant shortcomings due to the impersonal nature between students and the lecturer that sometimes leads to students frustration or disconnection from the educational environment [3,4]. In addition, traditional didactic lectures have serious educational limitations as they mainly improve the students’ ability to recall knowledge and do not suit higher levels of learning that require active participation and engagement of students within the educational environment [5,6]. Further, didactic lectures might not be the optimum mean to teach students who differ greatly in their cognitive skills and background knowledge. Nevertheless, as the lecture proceeds, the students’ attention, which has been reported to be around 15–20 min [7,8], wanes rapidly when the lecturer relies on just reading or explaining the information present on the PowerPoint slides projected onto a screen [9]. More recently, it was reported that the current generation learners have even shorter attention span that lasts 7 min [10] or less [11]. Another drawback of large lectures is the lack of instant feedback on students’ understanding or progress during information delivery [3].

Opposing didactic lectures where students just passively listening to the lecturer, active learning strategies address the educational contents in an interactive manner. As learning basically relies on what the learner does not the lecturer [3]. In active learning, the students perform instructional activities and think about what they are doing through reading, writing, discussing, or being engaged in solving problems [3]. This allows the students to reflect back upon what they have learnt and eventually produce significant improvement in terms of the learning outcomes. Ideal active learning environments are rich in instructional activities that engage students in higher-order thinking activities such as analysis, evaluation and synthesis to improve conceptual understanding, problem-solving and critical thinking in students [12,13,14].

## 2. Reported Techniques to Initiate Active Participation in Lectures

To mend the weaknesses of large lectures for more pedagogically fruitful environment, many lecturers have proposed different ways to promote active participation of students and reduce the complete dependence on traditional lecturing [15,16]. Typical techniques encompassed engaging the students in small group discussions during the lectures. Yet, limitations were reported for these methods, wherein it was tough to manage group discussion in large lectures and ensure active involvement of all students [17]. Henley and Oakley (1998) integrated group debates in lectures to improve student-student interaction. However, this approach might not be suitable for class size that exceeds 100 students and lasts for 1 h [18]. In other cases, the large lectures based courses were split into smaller-group courses encompassing discussion to foster active participation [19]. Yet, this approach often requires a complete curriculum review that is somehow beneficial, however, many lecturers are not in a position to do this and they do not have choice in the lecture size that they teach.

Recently, different studies have included technology-based approaches like personal response systems or clickers to engage and motivate students within the lectures [20,21]. Yet, financial barriers limit the adoption of these engaging pedagogies due to the unavailability of laptops or tablets for all students in public pharmacy colleges . It is worth noting that colorcolored cards can be used as a low cost and low tech replacement for clickers. Another published way to amend the dynamics of large lectures and widen students’ participation was by allowing them to tweet their questions to the lecturer via Twitter. However, the main drawback is that tweeting can divert students' attention away from the lecture and disrupt the learning process [22,23,24]. Not to mention that this method discourages the face-to-face (student/teacher and student/student) interaction and communication.

## 3. Description of the 5-Step Paper-Based Model

Under all conditions and regardless of the number of students in the lecture, any learning environment should provide an opportunity for deep understanding of the material for all students. Accordingly, lecturers are obliged to identify and implement viable methods of instruction for large lectures to optimize students’ involvement using the available resources either low-cost or expensive.

**Figure 1:**
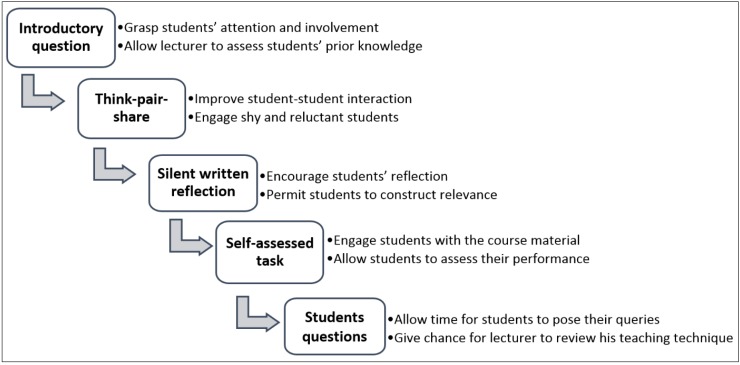
The main components of the 5-step paper-based approach.

In the current article, a simple 5-step paper based-model comprising various student centered activities is presented to initiate students’ engagement in large lectures. The main components of the 5-step paper-based approach are presented in Figure 1.

The key advantage to implementing this model is that it does notdoes not require any additional resources except photocopying the 5-step paper.

**Figure 2:**
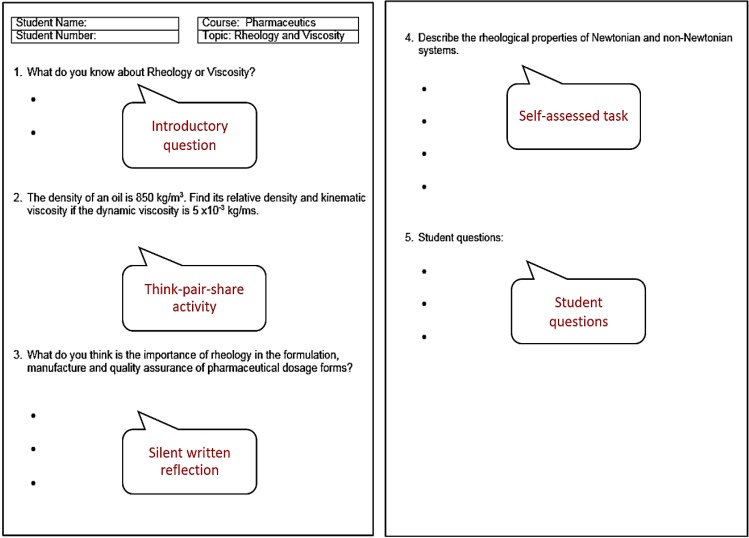
Sample outline for the 5-step paper-based model using a “Rheology and Viscosity” lecture delivered to pharmacy students.

A brief outline for the 5-step paper-based model using a “Rheology and Viscosity” lecture delivered for pharmacy students as an example is presented in Figure 2.

To create time for active learning in the lecture, the students were instructed to read and prepare the lecture before class to gain background information. At the beginning of the lecture, the 5-step papers are placed at the entry of the lecture hall so that students would take their papers while entering. To encourage them to take the process seriously and provide them with a sense of ownership, the students are required to write their names and respond to all the tasks on the 5-step paper at the set time. For each task, the students are committed to condense and present their ideas/responses in short written statements in the provided space. Writing is an integral component in this model to active engage students and keep them involved in tasks [25]. At the end of the lecture, the 5-step papers will ultimately be collected from the students although they are unassessed and do not count for points or grades in the course.

**Figure 3:**
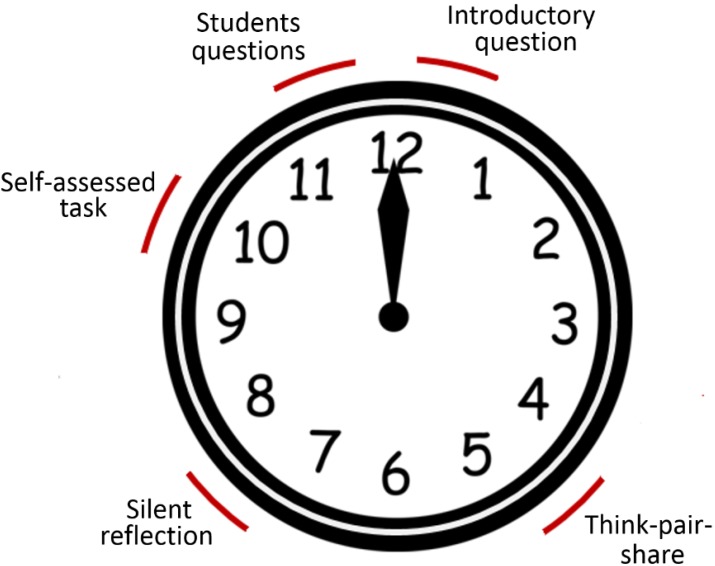
5-step paper-based model components dispersed during 1-hour lecture.

Based on the proposed model, the lecture is divided into approximately 3 or 4 lecture chunks/main points dispersed with preset writing tasks involving students discussion, reflection, and self-assessment to balance lecturing-style instructions with students-centered activities. The students’ tasks are integrated at certain timings during the 1-hour lecture as presented in Figure 3.

Such pacing by breaking down the 1 h lecture into short segments is essential to maintain students’ attention during the whole lecture by restarting their attention clock. After each task, lecturer feedback is given to students regarding their progress. A clear explanation of the components/tasks of the 5-step paper-based approach is presented hereafter:

### 3.1. Introductory Question

Incorporating an effective question at the beginning of the lecture is essential to reinforce students’ attention early on by challenging them to think and encouraging immediate participation [26,27]. The lecturer can also use this introductory question to determine the level of students’ prior knowledge for key concepts related to the lecture topic to build upon his new information [28]. Using this initially engaging approach, the lecturer brings the students “on lecture stage” with him/her instead of leaving them to passively hear the lecture. All the students are encouraged to write their responses on the submitted paper and few students are randomly selected to state their responses. It is worth mentioning that the lecturer ought to deal tactfully with all students’ contributions irrespective of the accuracy of the received responses.

### 3.2. Think-Pair-Share

In this section, the students are given a question, preferably a challenging one, to elicit higher level thinking. All the students are allowed to think then construct their responses individually, then, pair with the neighboring student to share their ideas followed by writing their response. Randomly selected pairs of students are given the opportunity to share their response in the lecture followed by lecturer feedback. The verbal articulation between the students in this activity offers the students, especially the shy or reluctant ones who find responding in large lectures intimidating, an opportunity to discuss and confirm their responses with peers resulting in more confidence and openness to share ideas. Educational researches indicated that peer discussions lead not only to improvements in students’ conceptual understanding and performance but also nurture greater involvement and self-confidence [29,30].

### 3.3. Silent Written Reflection

In this section, the students are given time to silently reflect on the key ideas presented in the lecture then seek their relevance or application in real life. The students are allowed to write their responses then randomly selected students share their ideas with the whole class followed by feedback from the lecturer. This activity helps the students to appreciate the relevance of the taught content to their future career which arise their interest and motivation in the subject content [31].

### 3.4. Self-Assessed Task

In this section, the students are given time to either summarize the main ideas delivered during the whole lecture or answer a comprehensive question in a pre-set time interval. After that, the lecturer presents the expected model answer and allow students to self-assess their written responses. Getting students to present their ideas in writing is a good approach for them to identify their misconceptions. This approach also assists the students in monitoring and evaluating the quality of their thinking and allow them to easily identify discrepancies between their current performance and the desired performance [3,32].

### 3.5. Student Questions

Finally, before the end of the lecture, the students are allowed to think, formulate and write down any questions they have. It is reported that students need time to actually reflect on previous instructions and articulate questions [33]. This simple practice of providing students adequate time to frame a question normally increase students’ contribution. Subsequently, that lecturer responds to the students questions.

At the end, the 5-step papers are collected from the students so that the lecturer would have the chance to gauge their participation and performance and address the unanswered questions, if present, in the coming lectures.

## 4. Advantages and Limitations of the 5-Step Paper-Based Model

This article describes a basic 5-step paper-based model that is based on a mixed-format to improve the pedagogy of large lectures in educational environment with limited resources.

The components of the 5-step paper based model are considered to emotionally and intellectually enhance interactions among students, the lecturer, and the lecture content using several tasks completed by students in writing.

In this model, the immediate feedback, whether to or from students, represents an essential element that caters for a two-way learning experience. Lecturer’s feedback helps the students in validating their understanding of concepts and apprehending their progress. In addition, students’ feedback give the lecturer an opportunity to evaluate students’ understanding and ascertain difficulty areas and adapts his teaching pace and methodology to meet their learning needs [34].

**Figure 4:**
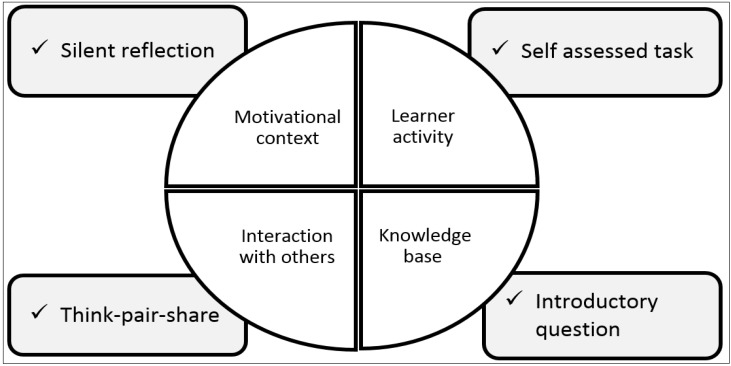
Mapping of 5-step paper-based model to Biggs four key features for deeper learning.

According to Biggs [35], four key components are essential in a learning process for students to achieve deeper learning: (a) Motivational context: where students are required to realize that the learning goals and processes are relevant to them, (b) Learner activity: where students are required to be active as deep learning is connected with doing rather than receiving passively, (c) Interaction with others: as discussions with peers promote students’ learning, and (d) Well-structured knowledge base: as new learning needs to be built on students’ prior knowledge and experience. Mapping of the presented 5-step paper-based model to Biggs four key features to achieve deeper learning is presented in Figure 4.

Table 1 summarizes the anticipated limitations that might be encountered when applying the 5-step paper-based model and types of solutions that may be employed.

**Table 1:** Issues that might arise during implementing the 5-Step paper based approach.

Problem	Potential Solution
Some students might find it hard to speak up in front of the whole class	Giving the student time to think about the question and then write their answer may be helpful.
	Think-pair-share activity allow shy students to present ideas to their partner giving them more confidence in their responses.
Some students are reluctant to participate in the activities	Allowing students to write their responses may motivate them to participate.
	Collecting the papers at the end of the lecture may facilitate tracking of non-participating students.
Off topic discussions between students during the think-pair-share activity	Short time limit- given for the think-pair-share activity decreases the chance for off-topic discussion.
High load for the lecturer to track students participation and questions posed from the collected papers	A quick scan of the papers can give an overview of the whole class participation in activities.
	Involving teaching assistants to compile the unanswered students’ questions. This might be a good learning excersise for them as it aquaints them with their future roles.
More time is consumed during the lecture than what was already anticipated for each task	Putting a tight time limit for each activity and limiting the writing space for each activity so that the students are concise in their replies.
Distribution and collection of papers at the beginning of the lecture and at the end is tedious and time consuming	Placing the papers at the entry of the lecture hall so that the studunts can collect them when they enter the lecture.
	Assigning students to collect the papers at the end of the lecture.
	The collected papers can be a good representation of students’ attendance in large lectures where their attendance is not usually tracked.
Not covering all the lecture contents as time is consumed in the students tasks	Assigning some topics for students to study on their own to promote their self-study abilities.
Lecturer time is consumed in preparing the 5-step paper based approach, redesign the lectures and realigning the self-study content	Initially, time is needed for lectures modification and designing the 5-step paper. However, once designed, they can be used for future classes with only slight modification if necessary.
Students may be distracted by the activities and attempt to read them before the lecturer permits	The paper may contain uncompleted questions that would be presented in the lecture slides at the preset time.
Photocopying is an additional expense	Providing small space for responding to each task so that the students are concise in their replies and not more than one paper is required for each student in the lecture.
Redundancy of the same presented format in all the lectures might decrease attention due to the loss of novelty.	Include other low cost active learning and collaborative activities that suit large lecture settings.

## 5. Implications for Implementing the 5-Step Paper-Based Model

In the 5-step paper-model, proper planning for how the content would be taught and ensuring that the students’ activities address the learning outcomes of the lecture is a necessity to creatively use the lecture time. The questions/tasks presented must be effectively designed to simulate deeper understanding and enhance pedagogical goals beyond just content memorizing [36].

The included students’ tasks should be specific to prevent students’ confusion. In addition, the lecturer should be strict with timekeeping of each task to efficaciously present and conclude the lecture content and discuss troublesome concepts within the allotted time. Collectively, the tasks of the 5-step paper-based model should not take more than 20 min of a 1 h lecture.

Although the lecturers are mainly held responsible for students’ learning, the recruitment of one or two teaching assistants in large lectures may be required to assist in reviewing the students’ questions section in the 5-step paper-based model, and to address the unanswered questions during the subsequent lectures or laboratory sessions to complete the feedback loop. It is of paramount importance to make sure that all the students’ questions are being recognized to motivate them to pose their queries in the coming lectures.

It is of no doubt that implementing active learning techniques takes time away from lectures, allowing insufficient time to complete the lecture content. In this context, different researches suggested that it is not essential to cover all the topics in the lectures. Some topics could be independently learned by the students to prepare them for their role as self-directed and lifelong learners [30].

For successful implementation of such approach in large lectures, it is advisable to delegate activities to different students in each lecture, like collecting and distributing the papers. In general, involving the students in the educational process might be helpful to develop their sense of responsibility and allow more intimate interaction with their lecturers.

## 6. Conclusions

Based on the anticipated increase in students’ enrollment in the public pharmacy colleges in Egypt, large lectures will continue to be the main method of teaching at least during the coming 10–15 years. Therefore, researches that investigate active and collaborative teaching methods that suit large lectures are paramount.

This article presents a simple 5-step paper-based model as an inclusive and engaging mean to increase students’ participation in large lectures in colleges with economic barriers. All that is required is the distribution of the 5-step paper to the students at the beginning of the lecture to engage them within the educational process using different writing tasks that involve students’ interaction, reflection and self-assessment, then collecting the papers at the end of the lecture. The presented model can be adapted or modified to suit individual lecture contents, course outcomes and teaching style. Although it is presented and discussed in terms of pharmacy discipline in this article, the 5-step paper-based model is relevant to teaching and learning in all disciplines. This model might also provide a valuable direction for lecturers to re-evaluate their routine in large lectures and serve as a basic tool to help them in adapting and ideally improving their lecturing technique.

## 7. Implications for the Future

This article presents a simple attempt to improve the teaching experience in large lectures instead of accepting its weaknesses. Moreover, the presented model might encourage lecturers who are wedged with large lectures to advance their lecturing practices and strive for excellence using available resources.

This paper-based model will be implemented in lectures for different courses in the pharmacy college to demonstrate its applicability. Both, unbiased lecturers’ perspectives along with students’ perspectives using different survey instruments that include questionnaires and open-ended questions in addition to pre- and post-intervention students test scores would be analyzed to assess the effectiveness of this model and give more realistic insight into its value and limitations.

## 8. Declaration

The views expressed in this paper are of the author and not an official position of the affiliated institute.
